# Odds and Ends

**Published:** 1859-10

**Authors:** Decatur P. Gregg


### ARTICLE III.

*Odds and Ends.* By Decatur P. Gregg, D. D. S.

If each member of the profession were to consult the interests of our art more, there would be less of that disposition exhibited to lay up for his own use, such improvements or suggestions as the benefits of experience have taught is useful. There are many items, when considered separately would hardly attract the attention for a moment, when aggregated, form, to no considerable extent, a culmination of practical values, which in the main constitute the science of dentistry itself. This much by way of preface, for want of either ability or time to give an essay on some interesting topic.

To those who have no disposition to peruse long theories of doubtful application or perhaps of equally unintelligible appreciation, I will dot down a few things experience has taught me, and the fastidious or critical may take down the whole, or appropriate it in broken doses, as an alterative, or otherwise, as their fancy or taste may dictate.

*Uses of Gutta Percha.*—Five years of constant use has satisfied me that one of the uses of that article is of much value in the readaptation of temporary sets of teeth, as well as when there is such a tenderness of the parts, that the hard metals will give pain. Let your "gutta percha" be rolled out to the thickness of common pasteboard, (not thinner,) heat up the plate (with a soldering lamp and mouth pipe) on the upper surface moderately, so that in applying the material it will soften and adhere firmly to the plate. You then can throw the flame on the under side of the plate, if you find it has not stuck well, or you can use any convenient instrument or pocket knife, and distribute it regularly over the plate by heating the blade in a spirit lamp.

After this is done, throw the flame upon the gutta percha so as to soften it, and apply it directly to a plaster or metallic cast, if you have them, taking care always to wet the casts beforehand to prevent the gutta percha from adhering to these casts. If you have no cast of the mouth, soften as before described, apply directly to the mouth, exactly in the same manner as if you were taking an impression, always taking care not to heat up so warmly as to burn the patient, which you can very easily determine by applying to the back of your hand—repeating the heating and application until a perfect coadaptation is secured. I have used gutta percha on my own plate of whatever kind, for these last five years. It will last from two to six months. It is easily renewed and is very comfortable, and amply compensates the operator in the satisfaction he gives his patient.

*Soldering Pans.*—The cheapest and best method for something to solder upon, is to take a large piece of well burnt charcoal, coat over *one* of the surfaces with plaster of paris, and make a handle of the same material, of convenient size and length—say five inches long and one in diameter, and you will have a better article to solder upon than all the improved pans or any thing else I have ever seen used. It is easily held, can be rotated conveniently whilst soldering. A large piece of pumice stone will do as well, though not so easily procured. Try it; and then if it suits you, use it—if not, throw it away. Small thing, say you—so it is—motes blind the eyes as well as larger substances.

*To Sharpen Files and Burr Drills.*—Great inconvenience as well as loss is sustained by not being convenient to dental depots in being unable to get good files or drills. Some may prefer making the latter, I would rather others would strain and damage their eyes than I mine by manufacturing them. Thousands of files thrown away as useless, may be made as valuable as ever with a cost of ten or twenty cents at most, with little or no labor, and consume only twenty minutes in doing it. Take your files and clean them well with brush, warm water and soap—procure some sulphuric acid—put in a vessel of convenient size, say a common glass tumbler, diluting it one-third with water, put in your files, let them remain ten, fifteen, or twenty minutes, which can easily be determined by taking one out and testing its sharpness. The length of time will depend upon the degree of dullness as well as the temperature of the acid. Care should be taken that none but the cut portion of the instrument be submerged in the liquor. Adopt the same method with your burr drills. I have some files in my possession I use every day, that I purchased six years ago, and I have sharpened them a dozen times. No one need try this who has any doubts about its value. I write for the benefit of those who are willing to learn and adopt any beneficial plan.

*To Temper Instruments.*—Procure an adamantine or sperm candle, heat the *points* of your excavators to a reddish color, stick them in the candle, and you will have a smooth, firm and elastic tempered cutting instrument as can be secured by all of the tedious and troublesome processes of drawing down the temper, as is too frequently taught.

*To support the Teeth while filling the Approximal Surface.*—Where there are spaces between the front teeth, it is sometimes painful to the patient to use the necessary pressure to condense a filling, I used for a long time pieces of soft wood, but lately I find a piece of India rubber for that purpose. Don't forget to try this—if you do you will be sorry for it. Now, if you think these odds and ends will be of any service, publish them.

